# The prevalence and costs of optical correction for childhood myopia in Scotland

**DOI:** 10.1111/opo.70007

**Published:** 2025-08-26

**Authors:** Emma Dow, Mhairi Day, Stephanie Kearney

**Affiliations:** ^1^ Department of Vision Sciences Glasgow Caledonian University Glasgow UK

**Keywords:** childhood, costs, myopia, prevalence

## Abstract

**Purpose:**

General Ophthalmic Services in Scotland (GOS(S)3) vouchers provide children with National Health Service (NHS)‐funded optical correction. This study investigated the financial impact of myopia by reporting current spending on GOS(S)3 vouchers and indicative prevalence of childhood myopia in Scotland.

**Methods:**

Data on the number and monetary value of GOS(S)3 vouchers issued for children aged 6–15 years in 2021/22 were obtained from Public Health Scotland. Data were categorised by age, refractive error and voucher value. Refractive error was defined as emmetropia (spherical equivalent refraction (SER) > −0.50D and < +2.00D), hyperopia (SER ≥ + 2.00D) and myopia (SER ≤ −0.50D). Total spending on GOS(S)3 vouchers and spending on additional GOS(S)3 voucher claims (due to spectacle prescription changes within 1 year) were reported. The proportions of additional GOS(S)3 vouchers for myopia and non‐myopic refractive errors (hyperopia and emmetropia combined) were compared with a two‐proportion *z*‐test. Indicative prevalence was estimated by dividing the number of GOS(S)3 vouchers (excluding additional vouchers) by the number of children in the population, using data from the National Records of Scotland.

**Results:**

Total annual spending on GOS(S)3 vouchers for the correction of emmetropia, hyperopia and myopia was £1.44 million, £1.43 million and £1.91 million, respectively. A greater proportion of GOS(S)3 vouchers was attributed to additional claims for myopia (6.31%) compared to non‐myopic refractive errors (4.10%) (*p* < 0.001). The prevalence of myopia as indicated by voucher claims ranged from 2.21% in children aged 6–7 years to 11.95% in 15 year olds. The proportion of children aged 6–15 years requiring spectacles for high myopia (spherical component < −6.00D) was approximately 0.26%, accounting for 3.62% of myopic children.

**Conclusion:**

Total spending on GOS(S)3 vouchers for the correction of myopia is lower than the combined spending on GOS(S)3 vouchers for emmetropia and hyperopia. However, children with myopia received more additional GOS(S)3 vouchers than other refractive groups, suggesting these children experienced more short‐term changes to their spectacle prescription.

**Key points**

- Myopia accounts for 40% of the annual spending on National Health Service spectacles for children aged 6–15 years in Scotland, at a total cost of £1.91 million per year.
- 6.31% of General Ophthalmic Services Scotland vouchers issued for myopia were due to changes in spectacle prescription within 1 year, compared to 4.10% for other refractive groups.
- Myopia affects 2.21% of 6–7‐year‐old children and 11.95% of 15‐year‐old children in Scotland. Approximately 0.26% of children aged 6–15 years have high myopia.

## INTRODUCTION

Myopia is a common refractive condition, which typically presents in school‐aged children due to an increase in the axial length of the eye.^1^, ^2^ The onset of myopia is generally followed by a period of progression, characterised by continued axial growth, until eventually stabilising in late adolescence or early adulthood.^1^, ^3^, ^4^ Whilst the refractive element of myopia can be corrected with spectacles or contact lenses, the associated increase in axial length predisposes the myopic individual to a number of sight‐threatening ocular complications in adulthood, such as myopic macular degeneration (MMD),^5^, ^6^, ^7^ rhegmatogenous retinal detachment (RRD)^8^, ^9^ and primary open angle glaucoma (POAG).^10^, ^11^ All myopes are at risk of developing these conditions; however, high myopia carries the greatest likelihood of complications.^12^, ^13^ In the UK, myopia is estimated to affect 12%–30% of children aged 12 years,^2^, ^14^, ^15^ but the prevalence of high myopia amongst UK children is unknown. The prevalence of childhood myopia in the UK has reportedly doubled since the 1960s,^2^ and further increases in myopia prevalence have been projected, with a recent paper predicting that myopia and high myopia will affect almost 40% of children and adolescents worldwide by the year 2050.^16^

The increase in myopia prevalence and its association with pathologies such as MMD, RRD and POAG has generated concern about the potential public health impact of myopia. Indeed, the development of myopia‐related diseases and subsequent visual impairment (VI) has a significant economic impact^17^, ^18^, ^19^ and can be detrimental to the quality of life.^20^, ^21^, ^22^ This has driven research into myopia control interventions, which aim to slow the rate of axial elongation in order to reduce the amount of myopia that accrues during childhood, thereby reducing the risk of disease and VI in later life.^23^ Interventions such as atropine,^24^ specialised spectacle lenses,^25^, ^26^, ^27^, ^28^ orthokeratology^29^ and dual focus contact lenses^30^, ^31^, ^32^ have demonstrated success in clinical trials at reducing the progression of myopia in children compared to single‐vision corrections. In addition to the potential health benefits, slowing myopia progression could lead to less frequent replacement of spectacles, and the resultant reduction in myopia severity may reduce the need for costly high index spectacle lenses. The unit price of myopia control is considerably higher than a traditional, single‐vision spectacle correction. However, the initial treatment outlay could be outweighed by the potential savings in future eyecare‐related costs and quality of life. Research conducted in Australia, China, Hong Kong, UK and France suggests that the correction of myopia with control interventions could be considered cost‐saving^17^, ^33^ or cost‐effective^34^, ^35^ over a lifetime duration.

In Scotland, a contribution towards the cost of spectacles or contact lenses is provided for children under the age of 16 years through the provision of General Ophthalmic Services Scotland (GOS(S)) optical vouchers, delivered by the National Health Service (NHS). Following an eye examination, if a child requires refractive correction, their optometrist can issue a GOS(S)3 voucher, which in most practices enables the child to receive a free pair of standard spectacles. This policy has been successful in providing children in Scotland with access to spectacle correction, regardless of socio‐economic background or location urbanity.^36^ GOS(S)3 vouchers can be used as a contribution towards the costs of optical myopia control interventions; however, the current voucher scheme does not cover the full cost of interventions. To date, the costs of providing NHS‐funded optical correction for children with myopia or other refractive errors have not been reported. Therefore, the aim of this paper is to explore GOS spending on the correction of refractive errors for the first time and report the indicative prevalence of childhood myopia and high myopia. The results of this study may contribute towards our understanding of the economic impact of myopia in the UK.

## METHODS

Data on the number and monetary value of GOS(S)3 vouchers issued in Scotland between 1st April 2021 and 31st March 2022 for children aged 6–15 years old were obtained from Public Health Scotland [https://www.publichealthscotland.scot].

GOS(S)3 vouchers provide a monetary contribution towards the costs of optical correction for all children under the age of 16 years and certain exempt adult patient groups in Scotland. Patients who meet these criteria are automatically issued a GOS(S)3 voucher if the prescribing optometrist recommends a change in refractive error or a change in spectacle frame/lenses following an eye examination.^37^ For children under the age of 16 years, a GOS(S)3 voucher is typically issued once per year alongside the child's annual eye test; however, an additional voucher can be issued if a change in prescription is required before then. There is no standardised threshold to define a change in refractive error; rather, the decision to issue an additional GOS(S)3 voucher is made on the basis of a clinically significant change, at the discretion of the prescribing clinician.^37^ The monetary value of the GOS(S)3 voucher varies depending on the complexity of the prescription, with the 2021/22 voucher values ranging from £39.10 to £215.50.^38^ If the patient desires spectacle frames or lenses which exceed the cost of the voucher, then the voucher can be used as a contribution to the spectacle cost and the parent or caregiver would be required to pay the remaining balance. Incidences of broken or lost spectacles that occur during the period in which the child is not due for an eye examination are covered by a different voucher scheme (GOS(S)4), which is not included as part of this study. All GOS voucher claims are linked to the patient's Community Health Index (CHI) number, a unique identifier, and are submitted electronically by practitioners to the NHS eOphthalmic database.

Two datasets were obtained from Public Health Scotland: one contained the total number of GOS(S)3 claims and a second dataset excluded additional claims. Additional claims refer to instances where more than one GOS(S)3 voucher was issued per patient per year; therefore, the latter dataset was used to estimate the number of children requiring refractive correction, to ensure patients with multiple GOS(S)3 voucher claims were not counted more than once. GOS(S)3 vouchers are assigned an alphabetical letter corresponding to their monetary value; the codes and voucher values for the financial year 2021/22 are provided in Table 1. Vouchers were categorised by year of age, refractive error group and voucher value. To maintain anonymity, the data were grouped and classified by staff from Public Health Scotland. A small number of data points were unavailable, to avoid identification of individuals due to low numbers within some categories. These data points were not included within the analysis.

**TABLE 1:** National Health Service (NHS) General Ophthalmic Services Scotland (GOS(S))3 voucher values during the study period 2021/22^38^ and present voucher values^39^ in UK Pounds Sterling.

Description	2021/22 voucher values	Present voucher values (from 1st April 2024)
A. Glasses with single‐vision lenses of a spherical power of not more than 6 dioptres with a cylindrical power of not more than 2 dioptres	£39.10	£42.40
B(a). Glasses with single‐vision lenses of a spherical power of more than 6 dioptres but less than 10 dioptres with a cylindrical power of not more than 6 dioptres	£59.30	£64.26
B(b). Glasses with single‐vision lenses of a spherical power of less than 10 dioptres with a cylindrical power of more than 2 dioptres but not more than 6 dioptres	£59.30	£64.26
C. Glasses with single‐vision lenses of a spherical power of 10 or more dioptres but not more than 14 dioptres with a cylindrical power of not more than 6 dioptres	£86.90	£94.14
D(a). Glasses with single‐vision lenses of a spherical power of more than 14 dioptres with any cylindrical power	£196.00	£212.40
D(b). Glasses with single‐vision lenses of a cylindrical power of more than 6 dioptres with any spherical power	£196.00	£212.40
E. Glasses with bifocal lenses of a spherical power of not more than 6 dioptres with a cylindrical power of not more than 2 dioptres	£67.50	£73.10
F(a). Glasses with bifocal lenses of a spherical power of more than 6 dioptres but less than 10 dioptres with a cylindrical power of not more than 6 dioptres	£85.60	£92.72
F(b). Glasses with bifocal lenses of a spherical power of less than 10 dioptres with a cylindrical power of more than 2 dioptres but not more than 6 dioptres	£85.60	£92.72
G. Glasses with bifocal lenses of a spherical power of 10 or more dioptres but not more than 14 dioptres with a cylindrical power of not more than 6 dioptres	£111.20	£120.48
H(a). Glasses with prism‐controlled bifocal lenses of any power or with bifocal lenses of a spherical power of more than 14 dioptres with any cylindrical power	£215.50	£233.56
H(b). Glasses with prism‐controlled bifocal lenses of any power or with bifocal lenses with a cylindrical power of more than 6 dioptres with any spherical power	£215.50	£233.56
I. (Hospital Eye Service) Glasses not falling within any of categories A‐H for which a prescription is given in consequence of a sight test by the Hospital Eye Service	£200.80	£217.58
J. (Hospital Eye Service) Contact lenses for which a prescription is given in consequence of a sight test by the Hospital Eye Service (the amount shown is per lens)	£57.00	£61.77

*Note*: All voucher values were included in calculations of the proportion of children requiring refractive correction for emmetropia, hyperopia and myopia. Only GOS(S)3 B(a), C and D(a) vouchers issued for myopia were used to estimate high myopia prevalence.

The sphere, cylinder and axis for each eye were provided on the GOS(S)3 vouchers by the prescribing clinician. Refractive error was defined as emmetropia (spherical equivalent refraction (SER) > −0.50D and <+2.00D), hyperopia (SER ≥ + 2.00D) or myopia (SER ≤ −0.50D).^2^, ^15^, ^36^ If a patient was myopic in one eye and hyperopic or emmetropic in the other eye, then they were counted as myopic.^15^, ^39^ If a patient was hyperopic in one eye and emmetropic in the other eye, they were counted as hyperopic. Patients were counted as emmetropic if they had emmetropia in both eyes. The number of GOS(S)3 B(a), C and D(a) vouchers (Table 1) issued for myopia was totalled to provide an estimate of the number of vouchers issued for high myopia. These voucher categories were chosen to represent high myopia as they are issued for single‐vision spectacle prescriptions with a spherical component < −6.00D (Table 1).

The total spending on GOS(S)3 vouchers and the spending on additional GOS(S)3 vouchers was reported for each refractive group. Additionally, the average cost of correction per child with refractive error was calculated for each refractive group by dividing the total spending on GOS(S)3 vouchers (including additional claims) by the number of children requiring refractive correction (as determined by the number of GOS(S)3 vouchers excluding additional claims).

Refractive error prevalence was calculated across the whole study population (all children aged 6–15 years), by year of age, and for two separate age cohorts: 6–7 and 12–13 years, to facilitate comparisons with previously published UK‐based studies.^2^, ^15^ Prevalence figures were calculated by dividing the number of GOS(S)3 vouchers, excluding additional claims, issued for each refractive category by the total number of children in the relevant age range living in Scotland (Equation 1). Age‐stratified population data were obtained from the National Records of Scotland.^41^ This method provides the indicative prevalence of refractive error, rather than true epidemiological prevalence. However, previous research using GOS(S)3 voucher data suggests that indicative prevalence calculated in this way aligns with values reported by epidemiological studies.^36^

(1) $$\begin{array}{c} \text{Refractive Error Prevalence}=\\ \frac{\text{Number of}\;\mathrm{GOS}\;3\;\text{vouchers}\;\left(\text{excluding additional claims}\right)\text{for each refractive group}}{\text{Number of children in population}} \end{array}$$

Equation 1: Calculation of indicative refractive error prevalence using GOS(S)3 voucher data.

Two‐tailed Pearson's *r* was used to explore the correlation between age and the number of singular GOS(S)3 voucher claims (excluding additional claims) for myopia, emmetropia and hyperopia, as the number of vouchers issued for each refractive category demonstrated a normal distribution (Emmetropia: *W* = 0.90, *p* = 0.22; Hyperopia: *W* = 0.94, *p* = 0.58; Myopia: *W* = 0.95, *p* = 0.65). To facilitate comparisons of the proportion of additional GOS(S)3 vouchers issued for myopia versus non‐myopic refractive errors, GOS(S)3 voucher data for emmetropic and hyperopic refractive groups were combined. The proportion of additional GOS(S)3 vouchers issued for myopia was compared to the proportion of additional GOS(S)3 vouchers issued for non‐myopic corrections using a two‐proportion *z*‐test. Statistical analyses were conducted using Jamovi [version, 2.3, jamovi.org], at the 5% level of significance.

## RESULTS

A total of 110,763 GOS(S)3 vouchers were issued by optometric practices in Scotland between 1st April 2021 and 31st March 2022 for children aged 6–15 years across all refractive groups. This figure includes 5,540 additional claims, where more than one claim was made per patient per year. As a result, 105,223 children aged between 6 and 15 years old received NHS spectacle correction in the year 2021/22, accounting for 17.6% of the Scottish population in this age range. Comparison of the datasets including and excluding additional claims suggests that 6.31% of the total number of GOS(S)3 claims for myopia were additional claims, compared to 3.64% and 4.62% for emmetropia and hyperopia, respectively. The proportion of GOS(S)3 vouchers attributed to additional claims for all non‐myopic refractive errors (hyperopia and emmetropia combined) was 4.10%. A two‐proportion *z*‐test indicated that the proportion of additional GOS(S)3 vouchers issued for myopia was significantly higher than the proportion of additional GOS(S)3 vouchers issued for non‐myopic refractive corrections (*z* = 16.58, *p* < 0.001).

### Spending on refractive correction

The total annual spending on NHS spectacle correction, including additional GOS(S)3 claims, was £4.78 million. Spending on the correction of myopia was £1.91 million, compared to £1.44 million and £1.43 million for the correction of emmetropia and hyperopia, respectively. In total, 6.36% of spending on myopia correction was attributed to additional GOS(S)3 claims. In contrast, the proportion of GOS(S)3 spending on additional claims for emmetropia and hyperopia was 3.71% and 4.60%, respectively. A summary of the spending on GOS(S)3 claims for each refractive category is provided in Table 2.

**TABLE 2:** Summary of the spending on General Ophthalmic Services Scotland (GOS(S))3 vouchers in each refractive group.

Refractive error group	Spending on GOS(S)3 vouchers excluding additional claims (£ thousand)	Spending on additional GOS(S)3 vouchers (£ thousand)	Total spending on GOS(S)3 vouchers (£ thousand)
Emmetropia	1382.4	53.2	1435.6
Hyperopia	1363.6	65.6	1429.2
Myopia	1789.0	121.6	1910.6

*Note*: Values are given in thousands of UK Pounds Sterling.

The average annual spending on GOS(S)3 vouchers per child with emmetropia, hyperopia or myopia was £42.61, £49.14 and £45.01, respectively. The combined average cost per child with either hyperopia or emmetropia was £45.63.

### Indicative prevalence of refractive errors

The proportion of children aged 6–15 years requiring optical correction for myopia, emmetropia and hyperopia is 7.09%, 5.63% and 4.86%, respectively. Approximately 0.26% of children were issued a GOS(S)3 voucher for high myopia, accounting for 3.62% of all myopic children.

**FIGURE 1:**
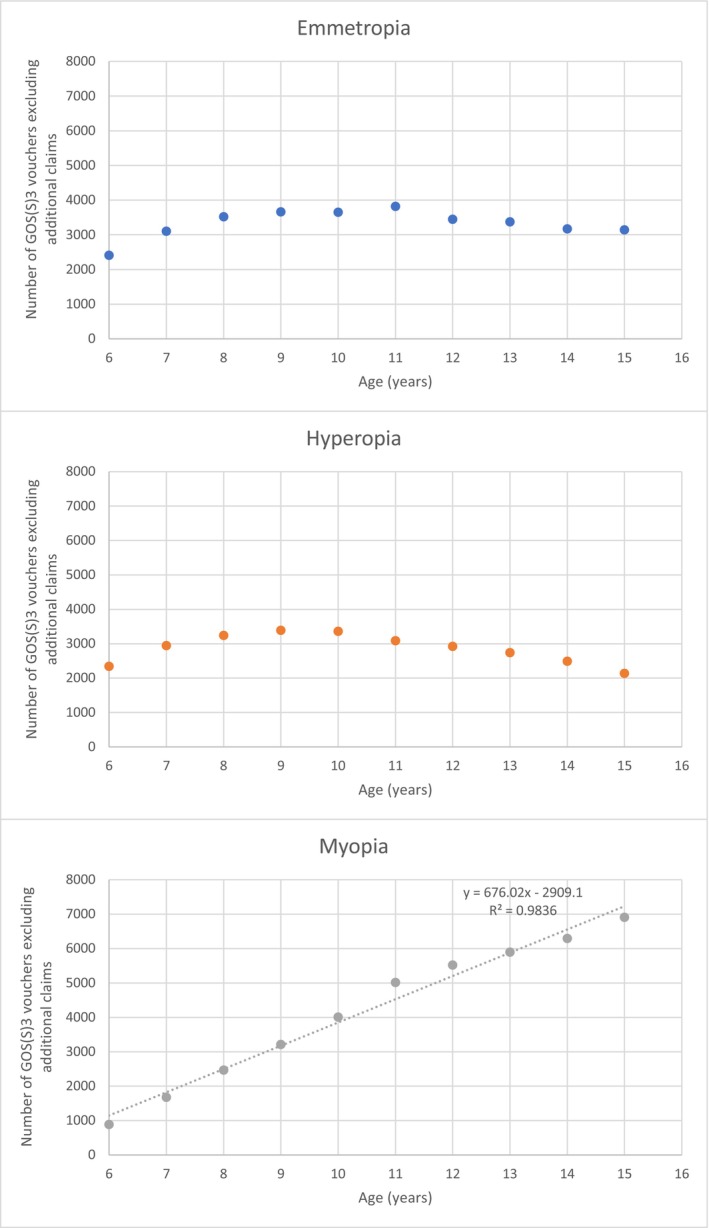
Scatter graphs illustrating the number of General Ophthalmic Services Scotland (GOS(S))3 vouchers (excluding additional claims) issued for emmetropia, hyperopia and myopia by age. The number of GOS(S)3 vouchers issued for myopia was significantly correlated with age (*r* = 0.99, *p* < 0.001). There were no significant correlations between the number of GOS(S)3 vouchers issued for emmetropia (*r* = 0.21, *p* = 0.59) and hyperopia (*r* = 0.39, *p* = 0.26) with age.

Table 3 provides a breakdown of the number and proportion of children requiring refractive correction for each year of age. The proportion of children aged 6–7 years who required an optical correction for myopia, emmetropia and hyperopia was 2.21%, 4.74% and 4.55%, respectively. In children aged 12–13 years, 9.29% required optical correction for myopia, 5.55% required correction for emmetropia and 4.61% required correction for hyperopia. By the age of 15 years, 11.95% of children in Scotland required optical correction for myopia, whereas 5.44% and 3.70% required correction for emmetropia and hyperopia, respectively. There was a strong positive correlation between the number of singular GOS(S)3 vouchers issued for myopia and increasing age (*r* = 0.99, *p* < 0.001) (Figure 1). The number of GOS(S)3 vouchers issued for emmetropia and hyperopia did not change significantly with age (*r* = 0.21, *p* = 0.59 for emmetropia and *r* = −0.39, *p* = 0.26 for hyperopia (Figure 1)).

**TABLE 3:** Number and proportion of children requiring refractive correction in the Scottish population, categorised by age.

Age, years	Population size	Children requiring refractive correction, *n* (%)		
		Emmetropia	Hyperopia	Myopia
6	57,945	2412 (4.16)	2341 (4.04)	890 (1.54)
7	58,262	3102 (5.32)	2947 (5.06)	1679 (2.88)
8	59,490	3522 (5.92)	3239 (5.44)	2467 (4.15)
9	60,960	3662 (6.01)	3392 (5.56)	3211 (5.27)
10	62,868	3653 (5.81)	3359 (5.34)	4009 (6.38)
11	59,950	3821 (6.37)	3091 (5.16)	5016 (8.37)
12	61,557	3448 (5.60)	2925 (4.75)	5524 (8.97)
13	61,334	3374 (5.50)	2739 (4.47)	5895 (9.61)
14	58,857	3174 (5.39)	2489 (4.23)	6294 (10.69)
15	57,792	3145 (5.44)	2136 (3.70)	6907 (11.95)

*Note*: Age‐stratified population data obtained from National Records of Scotland.^39^

## DISCUSSION

### Spending on refractive correction

A total of £1.91 million is spent annually on the provision of NHS‐funded optical correction for myopia in children aged 6–15 years in Scotland. This accounts for around 40% of the total spending on GOS(S)3 vouchers in this age range and 1.7% of the annual budget for all GOS services (free eye examinations and optical vouchers) provided by the Scottish Government.^42^ The majority of GOS(S)3 vouchers are issued for the correction of emmetropia and hyperopia, with a combined spend of £2.86 million per year, amounting to almost £1 million more than the cost of providing optical correction for myopia per year. The average annual cost of NHS‐funded optical correction per child with myopia (£45.01) is slightly lower than the corresponding cost per child with hyperopia or emmetropia (£45.63), which likely reflects a greater proportion of higher voucher values (corresponding to higher or more complex prescriptions) being issued for children with hyperopia.

Despite the overall greater expenditure on emmetropic and hyperopic refractive corrections, the annual spending on additional GOS(S)3 vouchers for myopia is greater than the equivalent cost for non‐myopic prescriptions. Indeed, 6.31% of the total number of GOS(S)3 vouchers issued for myopia were attributed to additional claims, compared to 4.10% for the correction of emmetropia and hyperopia, indicating that children with myopia experience more frequent changes to their spectacle prescription within a period of <12 months. Children with progressive myopia may be monitored on a shorter recall compared to those with other refractive error types, which could increase the likelihood of detecting prescription changes and subsequently contribute to the higher rate of additional GOS(S)3 claims. Myopia control may reduce the frequency of spectacle prescription changes by slowing the rate of axial growth and myopic progression. However, future research is required to evaluate this hypothesis and determine the effect, if any, this would have on spending on additional GOS(S)3 vouchers. A number of studies have explored the costs of myopia correction in China, Australia, Hong Kong and Singapore,^17^, ^35^, ^43^, ^44^ but these are not applicable to the UK and its publicly funded healthcare system. To date, there are only two studies, which reported the costs of myopia from a UK perspective,^33^, ^34^ both of which involved an economic analysis of myopia control interventions compared to traditional single‐vision correction. Lee et al. reported that myopia corrected with myopia control spectacles resulted in the lowest lifetime societal cost compared to single‐vision correction and other myopia control modalities in the UK.^33^ A cost‐utility analysis conducted by Health Technology Wales found orthokeratology and multifocal soft contact lenses to be the most cost‐effective options for managing myopia in Wales.^34^ The present study adds to the literature on the economic impact of myopia in the UK by providing an evaluation of NHS spending on optical correction for children with myopia and a comparison with other refractive error types to help contextualise the findings.

### Indicative prevalence of myopia

The prevalence of myopia, as indicated by GOS(S)3 voucher distribution, in children aged 6–7 years and 12–13 years living in Scotland is 2.2% and 9.3%, respectively, which is comparable to the results of prospective studies conducted in other parts of the UK. For instance, in the Northern Ireland Childhood Errors of Refraction (NICER) study, 1.9% of children aged 6–7 years were myopic at baseline, increasing to 14.6% by the age of 12–13 years.^2^ Similarly, the prevalence of myopia in the Avon Longitudinal Study of Parents and Children (ALSPAC) was 2.5% and 11.9% in children aged 7 and 12 years, respectively.^14^ Conversely, these prevalence figures are lower than those reported in the Aston Eye Study, which found that 9.4% of 6–7 year olds and 29.4% of 12–13 year olds were myopic.^15^ The increased myopia prevalence in the Aston Eye Study may be explained by the ethnic composition of the study populations. In contrast to the NICER and ALSPAC studies, which were conducted in predominantly White participants, the Aston Eye Study had a more diverse study population, with 50% of participants being of South Asian ethnicity.^15^ When the data from only White European children are examined, the prevalence of myopia in the Aston Eye Study aligns more closely with the results of the ALSPAC and NICER studies, with rates of 5.7% in 6–7 year olds and 18.6% in 12–13 year olds.^15^ Ethnicity information was not collected as part of the GOS(S)3 voucher submission process; however, it is assumed that the ethnicities of children receiving GOS(S)3 vouchers reflect the general ethnic distribution of the Scottish population (88% White in children aged 0–15 years^45^).

A linear relationship was observed between the number of children requiring refractive correction for myopia and age (Figure 1). Interestingly, within the literature, plots of myopia prevalence, progression or onset over time often include the use of cut‐off points,^4^ joining of data points^46^ or best fit curves,^47^, ^48^ with little information provided by authors to justify the choice. To determine whether a single best fit trendline, as opposed to multiple lines for specific age groups (e.g., 6–9 years, 9–12 years or 12–15 years), was most appropriate for illustrating the relationship between GOS(S)3 vouchers for myopia and age, the *R* ^2^ value and gradient was examined for all possible age combinations of three or more data points (e.g., 6–15 years, 6–14 years, 6–13 years…until 6–8 years; 7–15, 7–14, 7–13… until 7–9 years; 8–15, 8–14, 8–13…until 8–10 years, etc). The mean ± standard deviation *R* ^2^ value for all combinations was 0.99 ± 0.01, providing no strong justification to split the single trendline into sub‐sections. Although the prevalence of myopia is known to increase during childhood with increasing age,^49^ it would be expected that the prevalence rate starts to plateau in late adolescence, as axial elongation typically stabilises around this time.^4^ Consequently, the linear relationship observed in Figure 1 may be due to the age range considered in this study; we suspect that if data were available for later teenage years and early adulthood, then the number of persons with myopia would stabilise with increasing age.

### Indicative prevalence of high myopia

The prevalence of high myopia in children aged 6–15 years in this study is estimated to be 0.26%, which reflects 3.62% of children with myopia and 1.46% of all children requiring refractive correction. This figure is considerably lower than the prevalence of high myopia reported in East Asia. In Hong Kong, almost 2% of children aged 6–12 years have high myopia^50^ and 8.6% of children aged 6–18 years living in Beijing are highly myopic.^51^ The prevalence of childhood high myopia in the UK has not been reported previously in population‐based studies. A recent study conducted in a large London eye hospital found that high myopia accounted for 4%–6% of prescriptions issued for children under 17 years of age.^52^ Conversely, patients attending hospital clinics are likely to have additional ocular comorbidities or secondary causes of myopia; therefore, the findings may overestimate the prevalence of high myopia in the general paediatric population. Cases of high myopia in young children are often more complex than those that develop later in life as a result of ‘school myopia’ and may be associated with genetic systemic syndromes, disrupted visual development or prematurity.^53^ The findings of the present study suggest that high myopia affects only a small proportion of children living in the UK; however, as myopia can continue to progress beyond late adolescence,^4^, ^54^ it is expected that a greater proportion of these individuals will develop high myopia by adulthood. Indeed, previous studies have indicated that high myopia affects between 2.4% and 4% of UK adults.^55^, ^56^

The monetary value of GOS(S)3 B(a), C and D(a) vouchers issued for high myopia is greater to compensate for the increased cost of higher index lenses, often required for high prescriptions to improve spectacle comfort and cosmesis. In theory, a reduction in axial growth with myopia control may lower the likelihood of progression to high myopia and subsequently lead to reduced NHS spending on more expensive GOS(S)3 vouchers. However, as the prevalence of high myopia reported in this study is low, it is unlikely that myopia control would produce a substantial reduction in GOS(S)3 expenditure in these categories. Notably, myopia can continue to progress during late teenage years and early adulthood^4^, ^54^; therefore, the onset of high myopia may occur after the child has reached the age of 16, and by this age, only certain exempt groups are entitled to NHS‐funded spectacles. As a result, any potential savings in refractive correction spending achieved by lowering the magnitude of myopia may be more likely to benefit patients directly through the sale of private spectacles, rather than from an NHS perspective. However, reducing the proportion of children that develop high myopia by adulthood may reduce the prevalence of pathologies such as MMD, RRD and POAG, which is likely to result in greater NHS cost‐savings. Individuals that develop MMD, RRD and POAG are at risk of VI, which could have further financial consequences for the health and social care sector, including spending on low vision rehabilitation, community and residential care and welfare payments.^18^ Globally, VI from MMD is estimated to cost US$6 billion per annum due to productivity losses alone.^19^ In the UK, the total annual cost of sight loss and blindness amounts to £15.8–28 billion, which includes direct healthcare related costs and indirect costs such as low vision aids and rehabilitation, residential and community care, welfare payments, potential lost productivity from informal care requirements and reduced workforce participation.^18^ Notably, the proportion of spending on VI in the UK attributed to myopia‐related conditions is unknown.

### Considerations

Data on the number of GOS(S)3 voucher submissions from primary eyecare practitioners were used to estimate the prevalence of refractive errors and associated NHS spending on optical corrections. The GOS contract provides optometrists in Scotland with enhanced responsibilities for the management of eyecare within the community, and as such, they are the first port of call for eye‐health and vision‐related concerns.^57^ However, there may be a small number of children under the care of the Hospital Eye Service (HES) or private eye care practices who would not be accounted for using the present methods, potentially resulting in an underestimation of refractive error prevalence. Similarly, NHS spending on refractive correction for children under the care of the HES is not included in the GOS(S)3 voucher dataset. There are a number of additional areas of NHS spending associated with the correction of refractive errors, which were not accounted for in the present study, such as the costs of primary and supplementary eye examinations and the repair or replacement of spectacles, which are funded through GOS(S)1 and GOS(S)4 services, respectively.

Whilst cycloplegic autorefraction is considered the gold standard for measuring refractive error in research studies,^58^ the methods of refraction used in clinical practice are likely to differ. Cycloplegic agents are routinely used in the examination of children in Scotland; although optometrists will use their own clinical judgement as to whether a cycloplegic refraction is necessary for a given patient. When cycloplegia is not used, the resultant SER may be more negative than that measured under cycloplegic conditions. Indeed, in a study of 6825 children aged 4–15 years, the mean difference in SER between cycloplegic and non‐cycloplegic autorefraction was −0.63D (95% confidence interval (CI): −0.61 to −0.65D).^59^ Therefore, the absence of cycloplegia may have led to inconsistencies in the classification of refractive errors in the present study, possibly resulting in an overestimation of the number of children categorised as myopic and an underestimation of children with emmetropia and hyperopia.

When defining the refractive status of an individual, researchers must choose how to manage data from the right and left eyes. In this study, a child was classified as myopic if myopia was present in either one or both eyes. This method aligns with previously published research on refractive error prevalence in UK children.^15^, ^39^ However, other similar investigations have used alternative methods to categorise refractive error, such as the use of data from one eye only^2^ or the mean SER of the right and left eyes.^14^, ^55^, ^56^ Defining an individual as myopic based on the presence of myopia in either one or both eyes could bias the classification of refractive error towards myopia compared to the alternative methods.

It was noted that there was a substantial proportion of children requiring refractive correction in the emmetropia category. This is most likely due to the definitions of emmetropia and hyperopia used in this study. A number of symptomatic children with a low plus prescription may be prescribed a refractive correction, yet would be categorised as ‘emmetropic’ if their SER was between −0.25D and +1.75D. However, the refractive error definitions used in this study align with earlier epidemiological studies of refractive error prevalence in UK children^2^, ^15^ and were also used to categorise refractive error in a recent study using a similar dataset of GOS(S)3 vouchers.^36^ GOS(S)3 voucher data is a useful metric for estimating the prevalence of myopia in Scotland, and aligns well with myopia prevalence figures in similar age groups from other parts of the UK.^2^, ^14^ Yet, it is important to note that the proportion of GOS(S)3 vouchers issued in the emmetropia category does not represent the prevalence of emmetropia, as a GOS(S)3 voucher is issued only when an optical correction is required. Since 17.6% of children aged 6–15 years in Scotland were issued a GOS(S)3 voucher for optical corrections, 82.4% do not require refractive correction, which may better reflect the prevalence of emmetropia in this demographic.

The number of B(a), C and D(a) GOS(S)3 vouchers indicated the prevalence of high myopia. These voucher categories were chosen as the spherical element of the child's prescription must be <−6.00D. However, this classification differs from International Myopia Institute (IMI) guidance, which recommends that high myopia should be defined as a SER ≤ −6.00D.^60^ Practitioners are advised to complete prescription details on the GOS(S)3 form in a format which maximises the spherical value, to ensure that the correct voucher value is established.^38^ For an individual with myopia, the highest sphere is obtained when the prescription is written in plus cylinder format. Inputting refractive error data in this way means that a child with moderate‐high astigmatism may be classified as a high myope despite failing to meet the SER criteria set out by the IMI. As a result, the methods of defining high myopia used in this study may have overestimated the high myopia prevalence in children. On the contrary, as the study included only GOS(S)3 vouchers issued by optometrists working in community practices, and not HES vouchers, it is possible that the true number of children with high myopia has been underestimated.

Using GOS(S)3 vouchers as a proxy measure for prevalence relies on the assumption that those who need refractive correction will attend an optometric practice for a sight test. As a result, barriers to accessing eye care services could affect the prevalence figures reported in this paper. In Scotland, the GOS contract entitles all residents to receive free, NHS‐funded eye examinations. This policy has increased the uptake of eyecare services since its introduction in 2006; although differences in service utilisation have been observed between different socio‐economic groups, with the poorest uptake amongst those with low incomes and lower levels of education.^61^ However, a more recent paper has indicated a largely equal distribution of optometry practices across deprivation quintiles, and a high positive correlation between the share of practices and share of residents within each quintile.^62^ Furthermore, Kearney et al. found that the proportion of GOS(S)3 vouchers issued for children aged 6–15 years was largely similar across deprivation quintiles, with a slightly higher proportion of GOS(S)3 vouchers issued within the most deprived areas, providing evidence that the uptake of paediatric eyecare services in Scotland is not adversely affected by socio‐economic status.^36^ Generally, the number of NHS eye examinations undertaken in Scotland has gradually increased since the introduction of GOS legislation, from 1.57 million in 2006/07 to 2.26 million in 2022/23.^63^ Whilst the COVID‐19 pandemic reduced the number of GOS eye examinations conducted in 2020/21, by the financial year 2021/22, the number of GOS examinations had returned to pre‐pandemic levels (2.34 million in 2018/19 vs. 2.21 million in 2021/22).^63^ Therefore, it is not anticipated that the COVID‐19 pandemic affected the number of GOS(S)3 vouchers issued during the period of data collection.

### Conclusion

While the majority of spending on NHS optical corrections is for non‐myopic refractive errors, children with myopia require more frequent alterations to their spectacle correction, leading to greater spending on additional GOS(S)3 vouchers compared with other refractive groups. Future research should consider whether myopia control interventions could play a role in reducing the proportion of additional GOS(S)3 vouchers issued for myopia by reducing myopia progression and the frequency of spectacle prescription changes. The findings of this study indicate that the prevalence of high myopia in children is low compared to other parts of the world, with approximately 0.26% of children aged 6–15 years affected; therefore, reducing the risk of becoming highly myopic with myopia control is unlikely to result in a substantial reduction in GOS(S)3 voucher spending. However, it may result in healthcare savings elsewhere, such as a reduction in the overall costs of managing myopia‐related diseases and visual impairment. Further research exploring these costs would improve our understanding of the economic impact of myopia in the UK.

## AUTHOR CONTRIBUTIONS

**Emma Dow:** Conceptualization (supporting); formal analysis (lead); methodology (equal); writing – original draft (lead); writing – review and editing (equal). **Mhairi Day:** Conceptualization (supporting); formal analysis (supporting); methodology (supporting); writing – original draft (supporting); writing – review and editing (equal). **Stephanie Kearney:** Conceptualization (lead); formal analysis (supporting); methodology (equal); writing – original draft (supporting); writing – review and editing (equal).

## FUNDING INFORMATION

No external funding was obtained to conduct this research.

## CONFLICT OF INTEREST STATEMENT

The authors report no conflicts of interest.
